# Proline Metabolism in Tumor Growth and Metastatic Progression

**DOI:** 10.3389/fonc.2020.00776

**Published:** 2020-05-15

**Authors:** Cristina D'Aniello, Eduardo J. Patriarca, James M. Phang, Gabriella Minchiotti

**Affiliations:** ^1^Stem Cell Fate Laboratory, Institute of Genetics and Biophysics “Adriano Buzzati-Traverso”, CNR, Naples, Italy; ^2^Mouse Cancer Genetics Program, Center for Cancer Research, National Cancer Institute at Frederick, NIH, Frederick, MD, United States

**Keywords:** proline, metabolic reprogramming, PRODH, ALDH18A1, PYCR1, collagen prolyl-hydroxylases, epigenetic remodeling, Budesonide

## Abstract

Cancer cells show a formidable capacity to survive under stringent conditions, to elude mechanisms of control, such as apoptosis, and to resist therapy. Cancer cells reprogram their metabolism to support uncontrolled proliferation and metastatic progression. Phenotypic and functional heterogeneity are hallmarks of cancer cells, which endow them with aggressiveness, metastatic capacity, and resistance to therapy. This heterogeneity is regulated by a variety of intrinsic and extrinsic stimuli including those from the tumor microenvironment. Increasing evidence points to a key role for the metabolism of non-essential amino acids in this complex scenario. Here we discuss the impact of proline metabolism in cancer development and progression, with particular emphasis on the enzymes involved in proline synthesis and catabolism, which are linked to pathways of energy, redox, and anaplerosis. In particular, we emphasize how proline availability influences collagen synthesis and maturation and the acquisition of cancer cell plasticity and heterogeneity. Specifically, we propose a model whereby proline availability generates a cycle based on collagen synthesis and degradation, which, in turn, influences the epigenetic landscape and tumor heterogeneity. Therapeutic strategies targeting this metabolic-epigenetic axis hold great promise for the treatment of metastatic cancers.

## Introduction

Metastatic seeding of tumor cells to distant body sites relies on the extraordinary phenotypic plasticity of cancer cells (1, 2). The acquisition of cancer cell plasticity is emerging as the adaptive response to a hostile tumor microenvironment. A paradigm of cell plasticity is the epithelial to mesenchymal transition (EMT) by which epithelial cells acquire mesenchymal traits while losing epithelial-specific gene expression. This phenotypic switch occurs through a continuum of intermediated cellular states in which cells acquire intermediate/metastable phenotypes, adopting phenotypic, and molecular features of both epithelial and mesenchymal cell types (3, 4). How this multistep process is controlled is a key question and a major unresolved issue.

A central role for metabolism is emerging in the control/modulation of cancer cell plasticity (5). Upon the activation of oncogenic pathways, cancer cells undergo metabolic reprogramming, adapting their metabolism to the energetic and anabolic requirements necessary for uncontrolled proliferation and motility (5). For instance, cancer cells become dependent on an exogenous source of non-essential amino acids (NEAAs), which are involved in synthesis of macromolecules redox balance, and post-translational and epigenetic modifications, i.e., the NEAAs take on regulatory functions essential for malignant growth and metastasis. Thus, it has been hypothesized that during cancer progression, some NEAAs may become “conditionally essential,” however, it is not only the product amino acid but also the metabolic pathway, itself, which is important (6–8).

In this context, a great interest is emerging on the role of the extracellular matrix (ECM) as a readily available source of limiting metabolites and an important component of the tumor cell plasticity. ECM proteins are a great reservoir of amino acids, mainly NEAAs, that can be released in the tumor microenvironment by the activity of matrix metalloproteinases/collagenases secreted by cancer cells (9) and thus influence cancer cells metabolism. Among NEAAs, ECM proteins are particularly rich of Glycine and Proline, and the regulatory functions and the impact of Proline metabolism on normal and cancer cell behavior has been deeply investigated and well-described (10–12).

The goal of this review is to describe the emerging knowledge on the role of Proline metabolism, mainly Proline synthesis, in the control of cancer cell plasticity, the genes/enzymes/pathways involved and their relevance as prognostic markers and potential therapeutic targets. Finally, we will discuss the emerging idea that cancer cells epigenetic and phenotypic plasticity rely on a Proline-dependent cycle, based on collagen-synthesis and degradation, which represents a potential target for the future development of novel anti-cancer therapies.

## Proline Catabolism in Cancer

The conversion of Proline into Δ^1^- pyrroline-5-carboxylate (P5C) is the first step of Proline catabolism, and is catalyzed by Proline dehydrogenase/Proline oxidase (PRODH/POX) enzyme. During this enzymatic reaction, flavin adenine dinucleotide FAD is reduced to FADH_2_, which may be used to generate ATP through the oxidative phosphorylation process. PRODH enzyme is bound to the inner mitochondrial membrane and its overexpression, concomitantly with high levels of free Proline, may concur to generate reactive oxygen species (ROS). In a second oxidative step, Proline-derived P5C can be converted into Glutamate in a reaction catalyzed by the pyrroline-5-carboxylate dehydrogenase (P5CDH) enzyme. Glutamate, after conversion into α-Ketoglutarate (α-KG), can be burned to CO_2_ using the TCA cycle, gaining ATP. Thus, cells can use Proline to produce ATP, other metabolites (P5C, glutamate, αKG) and ROS. The role of PRODH-mediated Proline oxidation in the proliferation/survival of cancer cells has been exhaustively described elsewhere (11–14). Here we report a brief description of the contrasting effects (anti- vs. pro- tumor) of PRODH on cancer cell behavior.

### Antitumor

PRODH is a p53-induced gene and its expression is down regulated in many tumors (15–18), most likely those carrying inactivated/mutated p53 variants. PRODH expression is also induced by the inflammatory factor peroxisome proliferator-activated receptor gamma (PPARγ) and AMP activated protein kinase (AMPK), whereas it is repressed by oncogenes, such as MYC, which acts through miR-23b^*^ (19). Overexpression of PRODH gene in colorectal cancer cells blocks cell cycle and reduces DNA synthesis (20). PRODH-induced ROS are strong inducers of apoptosis and autophagy. PRODH activity can also concur to suppress hypoxia-inducible factor 1 alpha (HIF1α)-mediated signaling by increasing the synthesis of α-KG, in hepatocellular carcinoma (21, 22).

### Protumor

PRODH expression is induced under hypoxic conditions in different tumor cell lines and in a mouse xenograft model of human breast tumor, and contributes to cancer cell survival by inducing autophagy (23, 24). Moreover, PRODH is upregulated in a 3D spheroidal cell culture model of breast cancer (BC) compared to the 2D culture, as well as in metastases compared to primary tumors in BC patients (25) (Table 1). PRODH inhibition impairs spheroids growth and reduces lung metastases formation *in vivo* (25). PRODH/POX contributes to survival of triple negative breast cancer (TNBC) cells treated with HDAC inhibitors (Table 1). PRODH ablation reduces pro-survival autophagy and increases apoptosis induced by the HDAC inhibitors used (45). PRODH induces, *in vitro* and *in vivo*, non-small cell lung cancer (NSCLC) cells toward EMT, proliferation and migration, which are blocked by depletion of PRODH (46) (Table 1).

**Table 1:** Proline-related genes and associated cancer types.

	**Proline-related genes**										
**Cancer Type/Organ**	***ALDH18A1***	***PYCR1***	***PRODH***	***EIF5A***	***P4HA1***	***P4HA2***	***P4HB***	***LEPREL4***	***PLOD1/2***	***MMP9/1/13***	***ATF4***
Adrenal											
Bladder											
Brain											
Breast											
Cervix											
Colon											
Esophagus											
Gastric											
Germ Cell											
Head-Neck											
Hepatic											
Kidney											
Leukemia											
Lung											
Lymphoma											
Melanoma											
Myeloma											
Ovary											
Pancreas											
Prostate											
Thyroid											
Reference	(26–29)	(26, 27, 30) (28, 31) (32–44)	(15–18) (21–25, 45, 46)	(47)	(48) (49–56)	(57) (58–65) (66, 67)	(68–72)	(26)	(26)	(26)	(73, 74)

*The gray boxes indicate that the specific gene has been associated to the specific cancer type*.

All together these findings support the idea that the pro- or anti-survival roles of PRODH in cancer cells may be context/environment- and cell type- dependent (75). Additionally, the product of PRODH activity P5C is the immediate precursor of Proline. The Proline-P5C cycle provides unique functions in amino acid metabolism (14).

## Proline Biosynthesis Genes Predict Poor Prognosis in Cancer

*De novo* synthesis of Proline is supported by Glutamine-derived Glutamate. In a first step, the P5C synthetase enzyme, encoded by aldehyde dehydrogenase 18A1 (ALDH18A1) gene catalyzes the conversion of Glutamate to P5C. In a second reductive step, P5C is converted to Proline by P5C reductase (PYCR) enzymes (10). Three isoforms (PYCR1, PYCR2, and PYCRL) of P5C reductase, each with distinct properties, have been identified (76). PYCR1 and 2 share a high amino acid (aa) sequence similarity (84%), they are both located in the mitochondria and prefer NADH as electron donor. Conversely, PYCRL shares only 45% of the aa sequence similarity with PYCR1 and 2, is localized in the cytosol and preferentially uses NADPH as reducing agent. PYCR2 is more sensitive to feedback inhibition by Proline (Ki ~0.15 mM) than PYCR1 (Ki ~1.0 mM), whereas PYCRL appears insensitive to Proline inhibition (10, 14). Of note, the up regulation of Proline synthesis from Glutamine by cMYC (77), and NAD^+^ NADP^+^ produced during Proline synthesis are potent regulators of both glycolysis and the pentose phosphate pathway, strongly suggesting its importance in cancer (8).

The role played by PYCRs-mediated Proline synthesis in cancer progression is supported by unbiased transcriptomics, metabolomics, and proteomics studies, indicating that PYCRs expression levels, especially PYCR1, influence the clinical course of cancer (Table 1).

A comprehensive study comparing the mRNA expression profiles of 1,454 metabolic enzymes across 1,981 tumors covering 19 different tumor types vs. 931 matched normal tissue controls, identify Proline biosynthesis genes (PYCR1 and ALDH18A1) among the most up regulated enzymes (26). The Cancer Genome Atlas (TCGA) database and gene expression profiles from a Singapore-based cohort reveal that PYCR1 and ALDH18A1 are among the most up-regulated genes in Hepatocellular Carcinoma (HCC). They both correlate with HCC grade, and predict a poor clinical outcome (27). PYCR1 knock-down (KD) cells show decreased cell proliferation, and a reduction of the NAD^+^–induced glycolytic and NADP^+^–dependent oxidative pentose phosphate pathways has been suggested (27). An independent study reveal that PYCR1 is induced in HCC tumor tissues compared to adjacent normal liver tissues and, remarkably, that PYCR1 ablation induces apoptosis, decreases cell proliferation, colony formation ability *in vitro*, and reduces *in vivo* tumor size (30). Moreover, a link between PYCR1 expression and activation of c-Jun N-terminal kinase (JNK) and insulin receptor substrate 1 (IRS1) signaling has been also suggested (30). Different studies reported that ablation of PYCR1 generates smaller tumors. However, besides reduced proliferation/cell number and/or increased apoptosis, lower tumor volume can be the consequence of reduced stroma/ECM. Indeed, lower levels of Proline affect collagen/ECM accumulation, which eventually results in smaller/more compact tumors that have less capacity to invade and generate metastasis (57).

In Breast Cancer (BC) tumors, PYCR1 and ALDH18A1 expression levels varies among specific BC subtype. An increase in PYCR1 copy number and PYCR1 mRNA level is associated with Luminal B type. Moreover, ALDH18A1 and Glutaminase protein levels are higher in high proliferative estrogen receptor positive (ER^+^) /human epidermal growth factor receptor negative (HER2^−^) (Luminal B) compared to low proliferative ER^+^/HER2^−^ (Luminal A) tumor cells, thus suggesting that the Glutamine-Proline axis is a poor prognosis marker in BC (28). By combining *in vitro* studies using BC cell lines and clinical data from human samples, Ding et al. found that PYCR1, but not PYCR2, is highly expressed in BCs independently of the specific subtype (ER^+^ vs. ER^−^), and positively correlates with tumor size, grade and invasiveness. Accordingly, PYCR1 KD reduces BC cells proliferation and invasiveness and increases the cytotoxicity of chemotherapeutic drugs, thus suggesting that PYCR1 may be a potential therapeutic target for BC (31). Complementary to these findings, Liu et al. developed a tool to calculate electrons energy dissipation during metabolic transformations (29), and found that under hypoxic conditions in which the electron transfer chain (ETC) to oxygen is blocked, proliferating cells rewire their metabolism and use Proline biosynthesis and lipogenesis as alternative electron acceptors. Blocking simultaneously ALDH18A1 and lipogenesis inhibits breast tumor growth *in vivo* and *in vitro* (29). A recent study demonstrates that infection with oncogenic Kaposi's sarcoma-associated herpesvirus (KSHV), an etiological agent of Kaposi sarcoma, increases Proline synthesis in a 3D model of breast cancer. KSHV K1 oncoprotein interacts with and activates PYCR1, promoting tumor growth and development. Abrogation of PYCR1 abolishes the oncogenic activity of KSHV K1 protein (32).

PYCR1 is highly expressed also in prostate cancer tissues (33), in renal cell carcinoma (RCC) (34), in papillary renal cell carcinoma (PRCC) (35) and in human malignant melanoma (MM) (36). These studies showed that PYCR1 expression strongly influence cell behavior (proliferation, colony formation, apoptosis) in different cancer contexts, and correlate with poor outcome and decreased overall survival in patients. Of particular interest is the finding that PYCR1 ablation inhibits migration and invasion both in PRCC and MM cells, altering phosphorylation of AKT (36) and mTOR (35).

Independent studies bring to the findings that PYCR1 is overexpressed also in non-small cell lung cancer (NSCLC) and has been associated with poor prognosis in patients with NSCLC (37–40). Knocking down PYCR1 inhibits NSCLC cell proliferation and cell cycle (37). Interestingly, PYCR1 expression is negatively regulated by *miR-488*, which inhibits cell proliferation and clone formation ability, and promotes apoptosis. These effects are rescued by PYCR1, which in turn activates p38 MAPK pathway (38). PYCR1 was shown to regulate NSCLC cell migration and invasion, and the expression of the typical epithelial-mesenchymal transition markers E-cadherin, Vimentin, N-cadherin, and Snail1, suggesting that PYCR1 may be critical for NSCLC aggressiveness and a potential target for treating NSCLC (39). Accordingly, lung adenocarcinoma cell sensibility to cisplatin increased upon PYCR1 silencing, further supporting the idea that PYCR1 is a potential therapeutic target for lung adenocarcinoma (40). Finally, recent findings showed a correlation between cancer cells that have mutation in isocitrate dehydrogenase 1 (IDH1) and increased PYCR1 expression and Proline levels (41).

Altogether, these findings further support a critical role of Proline biosynthesis genes in cancer development and progression, independently of the tumor type.

### Transcriptional and Post-translational Regulation of PYCR1 and ALDH18A1 in Cancer

Recent studies on the role of Proline biosynthetic enzymes in neuroblastoma (NB) progression unravel a novel mechanism of transcriptional regulation of the genes coding for these enzymes (42). Specifically, myeloid zinc finger 1 (MZF1) and MZF1 antisense RNA1 (MZF1-AS1) have been identified as transcriptional regulators of ALDH18A1 and PYCR1. MZF1 induces the expression of ALDH18A1 and PYCR1, promoting NB aggressiveness. Mechanistically, MZF1AS1 promotes the up-regulation of both MZF1 and of other oncogenic genes through the interaction with poly(ADP-ribose) polymerase 1 (PARP1), thus facilitating its interaction with E2F transcription factor 1 (E2F1). Interestingly, MZF1, MZF1AS1, PARP1, and E2F1 are all associated with poor prognosis of NB patients, and blocking MZF1AS1 and PARP1 interaction, using a small peptide, or targeting MZF1AS1 suppresses Proline synthesis and tuomorigenesis, thus representing potential targets for NB therapy (42).

Recently, a lncRNA *TRPM2-AS*/*miR-140-3p*/PYCR1 axis has been described in BC, which regulates cell proliferation and apoptosis. Specifically, while TRPM2-AS and PYCR1 are both overexpressed in BC, *miR-140-3p* is downregulated and directly targets both TRPM2-AS and PYCR1 (43).

To date the knowledge on PYCR1 post-translational regulation is still poor. Recently, it has been shown that SIRT3, a mitochondrial NAD^+^-dependent deacetylase involved in the regulation of several metabolic pathways, interacts with PYCR1 both *in vitro* and *in vivo*. Acetylation of PYCR1 at K288 residue by cAMP response element- binding protein (CREB binding protein, CBP) acetylase reduces its activity and leads to inhibition of cell proliferation. These findings link Proline metabolism with SIRT3 and CBP and cell growth, and suggest that this axis may be a potential target for cancer therapy (44).

Finally, expression of P5CS, PYCR1/2/L is increased by c-MYC and PI3K signaling in luminal B breast cancer (28) as was previously shown in cultured cancer cells after ectopic expression of c-MYC (77).

## Proline Availability Controls Cancer Cell Behavior

The emerging evidences that increased PYCR1 and ALDH18A1 expression is a poor prognosis factor in different tumor types are robust and convincing, and suggest an increased need of Proline biosynthesis in cancer cells. Proline can be used by cancer cells as energy source and/or as precursor of protein synthesis. An interesting finding regarding the requirement of Proline for proteinogenesis has been reported by Loayzcha-Puch and colleagues (78). The authors developed a protocol to measure differential ribosome codon reading (diricore), which is based on ribosome profiling measurements. The study reports a striking contrast between the diricore of cancer and normal surrounding kidney tissues and provides evidence for a cancer cells-specific limitation of Proline-tRNA availability for protein synthesis (78). These findings are complemented by the observation that PYCR1 gene is induced in cancer cells, likely as a compensatory feedback mechanism against a condition of Proline shortage. This can be provoked either by a reduced availability of exogenous Proline and Proline metabolic precursors (Glutamine and Glutamate), by a sudden increase of Proline consumption for proteogenic and energetic purposes and/or by both, i.e., an increased requirement with a reduced availability. The requirement of PYCR1 activity for tumor growth, further support the idea that Proline availability controls cancer progression (78). Interestingly, Proline restriction is not an unique feature of kidney cancer cells, but also of breast cancer cells (78), thus raising the possibility that it may be a common feature of different tumors types. Accordingly, some cancer cell lines are starved of Proline and depend on exogenous Proline to restore their clonogenic potential, and resolve endoplasmic reticulum (ER) stress (79).

**Figure 1:**
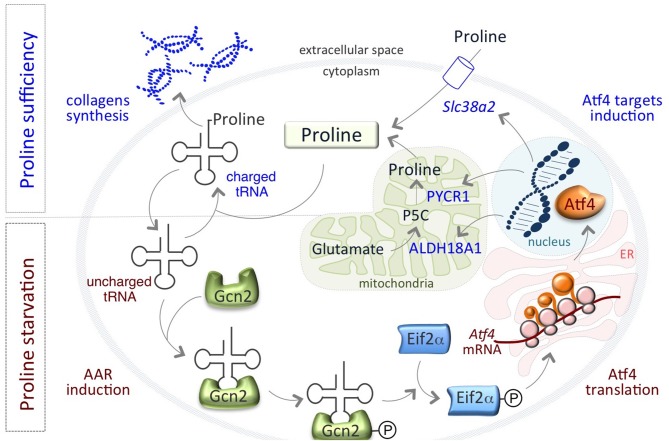
An autoregulatory loop controls Proline biosynthesis. Proline biosynthetic enzymes, ALDH18A1 and PYCR1 are under the control of the amino acid starvation pathway (AAR-ATF4). Under Proline limiting conditions, uncharged tRNAs accumulate and activate/phosphorylate Gcn2-eIF2α inducing the expression of the transcription factor ATF4, which in turn activates the expression of PYCR1, ALDH18A1, and the amino acid transporter *Slc38a2/SNAT2* genes. When Proline levels increases, tRNAs are charged and AAR-ATF4 pathway is relieved.

Remarkably, a finely regulated growth-limiting starvation of Proline is also a feature of mouse pluripotent embryonic stem cells (ESCs), which is required to preserve ESC identity. We have recently showed that the amino acid stress response (AAR)-ATF4 pathway, which is sensitive to nutrient starvation through the presence of uncharged tRNAs, is active in ESCs and controls the expression of the Proline biosynthesis genes *Aldh18a1* and *Pycr1* (80) (Figure 1). This generates an autoregulatory loop by which ATF4 induces Proline synthesis/accumulation, which in turn down regulates ATF4 through the relief of the AAR pathway (Figure 1). Interestingly, also the amino acid transporter *Slc38a2/Snat2*, which madiates Proline uptake in ESCs (81), is an ATF4 target gene (82, 83) (Figure 1). This autoregulatory loop causes a specific shortage of Proline that preserves ESC behavior/identity. When Proline levels suddenly increase, for instance as a consequence of Proline supplementation, the prolyl-tRNA is loaded and the synthesis of Proline-rich proteins, such as collagens, is induced (80). Consequent to the rapid increase of Proline availability, mESCs undergo a phenotypic transition named embryonic-stem-to-mesenchymal like transition (esMT). During esMT, the cells acquire mesenchymal-like, motile and invasive features, resembling the main characteristics of the migrating cancer cells (80, 84). Remarkably, pharmacological blocking of the prolyl-tRNA synthetase activity with the specific inhibitor halofuginone (85, 86) antagonizes Proline-induced esMT (80). These findings, together with the observations that the prolyl-tRNA synthetase-coding gene (26) and the transcription factor ATF4 (73) are overexpressed in different tumor types, and promote cancer metabolic homeostasis and survival (74) (Table 1), point to the key role of Proline availability in regulating protein synthesis, which sustains cancer cell proliferation and preserves stem cell identity. Tumor cells undergo metabolic reprograming that may restrict the availability of specific amino acids. Thus, sensing of amino acid availability by different pathways, including mTOR and AAR-ATF4 pathways, is crucial for cancer development, mainly by controlling the efficiency of protein synthesis and in particular of Proline-rich protein such as collagens.

## Collagen-Prolyl Hydroxylation in Cancer Progression

Tumor microenvironment and remodeling of the extracellular matrix (ECM) have gained increasing interest as mechanisms that contribute to the development of many solid tumors and in particular, to the reprogramming of cancer cells, conferring them with aggressive features, e.g., invasiveness, ability to undergo EMT and therapeutic resistance (87–89). Collagen is the most abundant protein in the human body and is the principal component of ECM. In this view, collagen biosynthesis, and maturation play a critical role. About 25% of the amino acids in collagen are incorporated as Proline and half of them are hydroxylated by a group of Fe^2+^ αKG/VitC- dependent dioxygenases, the prolyl-4- hydroxylase (P4H), within the endoplasmic reticulum (ER) (90). Hydroxylation of Proline residues is a critical modification required to stabilize the triple helix of collagens. It is thus reasonable to expect that incorporation of Proline into collagen may have metabolic consequences. P4H enzymes are tetramers made of 2α catalytic subunits and 2β subunits, and the genes encoding for these enzymes have been implicated in cancer progression (Table 1). High levels of P4HA2 correlate with poor prognosis in glioma, and P4HA2 knockdown blocks glioma cells proliferation, migration and acquisition of EMT-like phenotypes (58). Overexpression of P4HA2 has been implicated also in cervical cancer (59); papillary thyroid cancer (60); B-cell lymphoma (61); oral cavity squamous cell carcinoma development and recurrence (62, 63). P4HA2 is correlated to liver fibrosis and hepatocellular carcinoma (HCC) (64). Interestingly, TCGA database analysis reveal higher levels of P4HA2 in HCC patients with a shorter overall survival and a higher cancer grade (65). P4HA2 is thus emerging as a potential target for the development of novel therapeutic anti-cancer strategies. Elevated P4HA2 and 1 predict patient mortality in human breast cancers (48, 66). Moreover, prolyl hydroxylase gene expression is induced by hypoxia and promotes invasiveness and lung metastasis (48). Conversely, depletion of P4HA2 inhibited BC cell proliferation and invasiveness *in vitro* and *in vivo*, by reducing collagen deposition (66). P4HA2 has been recently reported to play a role in the progression of breast ductal carcinoma *in situ* (DCIS) (67). Furthermore, high levels of P4HA2 has been associated with decreased survival in a breast cancer dataset with almost 2000 patients, and is an independent predictor of disease outcome with respect to standard clinopathological parameters (57). Accordingly, silencing of P4HA2 converted Triple Negative Breast Cancer (TNBC) cells to a more epithelial phenotype, and reduces invasiveness in a 3D organotypic culture (57).

Deregulated P4HA1 expression has been also implicated in cancer development and progression. P4HA1 expression is induced in TNBC and correlates with short relapse-free survival in patients treated with chemotherapy (49). The authors show that P4HA1 promotes HIF1α-dependent cancer stemness and chemoresistance by reducing the availability of α-KG, and support the idea that P4H is a promising target to inhibit tumor progression and sensitize TNBC to chemotherapy (49). Elevated P4HA1 expression was recently described in pancreatic ductal adenocarcinoma (PDAC) and predicts poor prognosis. Of note, the authors found a P4HA1-HIF1α positive feedback loop, which regulates the glycolytic and oncogenic activities of PDAC, their stemness and chemoresistance (50). Additionally, high P4HA1 expression is a poor prognostic factor for head and neck squamous cell carcinoma (51), oral squamous cell carcinoma (52), and prostate cancer (53). In ovarian cancer P4HA1 promotes migration, invasion, EMT and metastasis formation (54). In glioma, P4HA1 promotes the transdifferentiation of glioma stem cells into endothelial cells leading to the formation of vascular basement membranes (55) and has been considered as a prognostic marker for high-grade glioma (56).

Prolyl-4 hydroxylases beta polypeptide (P4HB) is the beta subunit of P4H and belongs to the family of protein disulfide isomerase (PDI), which acts as chaperone in the ER to inhibit the aggregation of misfolded proteins. P4HB is overexpressed in different types of tumors, such as hepatocellular carcinoma (68), non-small-cell lung cancer (69), and in gastric cancer (GC) for which it has been considered to have a prognostic value (70). High levels are mainly associated with invasiveness and lymphatic metastases of cancers (71). Zhang et al. suggested a correlation between hypoxia/hypoxic microenvironment, which plays critical roles in the process of EMT, and P4HB in the context of GC. Specifically, HIF-1α up-regulates P4HB expression in gastric cancer and together they cooperate to promote GC invasion and metastases (72).

In line with the idea that collagen hydroxylation and maturation underlie tumor progression and metastasis formation, PLOD1/2 and LEPREL4 genes, which are both involved in collagen biosynthesis, are upregulated in 19 different tumor types, (26) (Table 1).

Interestingly, recent phosphoproteomic analysis reveal that P4HA2 and P4HB are targets of the tyrosine kinase PKDCC/VLK, which phosphorylates a broad range of secreted and ER-located proteins (91). Of note, PKDCC/VLK is one of the most up regulated genes in Proline-induced esMT (80, 84).

In most cases, P4H expression has been linked to the acquisition of mesenchymal/invasive features and metastasis formation. In particular, (i) the accumulation/deposition of collagen near tumors has been associated with metastasis formation, (ii) inhibition of P4H reduces tumor aggressiveness both *in vitro* and *in vivo*. Despite extensive studies, the mechanisms underlying P4H-dependent tumor aggressiveness are still poorly understood.

## Collagen-Epigenetic Interplay in Cancer Cell Identity and Plasticity

Increased collagen synthesis and maturation may in turn influence cancer cell growth and behavior by acting at different levels. The most studied role of collagen processing in tumors development/progression is focused on its downstream signaling. Different signaling pathways control synthesis/accumulation of collagens, including TGFβ. In the context of TGFβ-dependent activation of fibroblast and consequent production and secretion of matrix protein and wound healing, TGFβ promotes Proline biosynthesis in a SMAD4-dependent manner, to support collagen production (92). Collagens interact with specific receptors, e.g., discoidin domain receptors (DDRs) and integrins, and in turn activate downstream pathways including ERK, PI3K/AKT -NFkB, Focal adhesion kinase (FAK) and enhance migration and promotion of EMT. This complex, context- specific role of collagen signaling in cancer development and progression has been extensively investigated and recently reviewed (93).

It is now evident that signals from the microenvironment influence cancer cell behavior, and that tumor microenvironment, including ECM composition, largely influences tumor progression. Indeed, the changing in the ECM mechanical properties, the increased collagen deposition and stiffness influence cell plasticity, endowing cancer cells with elevated invasiveness, migration and metastatic dissemination properties (87–89). In this context, it has been recently shown that epigenetic silencing of the tumor suppressor RASSF1A in lung cancer induces P4HA2 expression, which leads to increased collagen deposition, ECM stiffness and triggers metastatic dissemination (94). An interesting interplay between Proline metabolism/collagen synthesis and microenvironment has been reported in a recent study on lung cancer, where the authors show the interaction of PYCR1 in the mitochondria with Kindlin-2, a protein critical for integrin-mediated cell-ECM adhesion. When ECM stiffness rises, as in cancer, Kindlin-2 translocates in the mithocondria where it interacts with PYCR1, increasing PYCR1 and proline levels. Kindlin-2 KD reduces PYCR1 levels and ECM stiffening-dependent increase of Proline synthesis. *In vivo* Kindlin-2 ablation strongly reduces PYCR1 and Proline levels, fibrosis, tumor growth and mortality rate (95).

**Figure 2:**
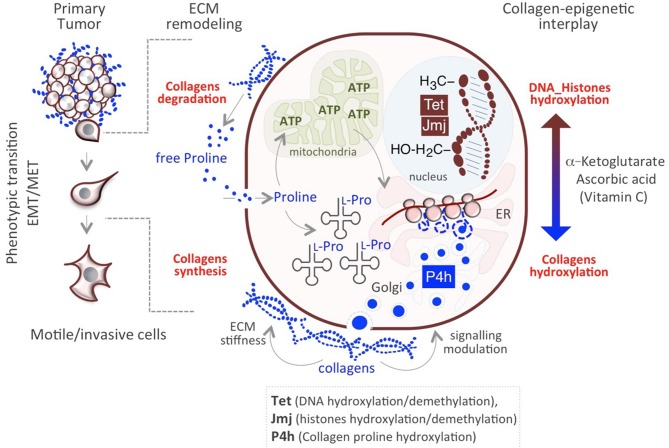
Impact of Proline/Epigenetic axis on tumor progression. Extracellular Proline released from collagen degradation influences cell identity/behavior at different levels. Proline may serve as (i) energy source (ATP) through degradation in the mitochondria and (ii) building block for collagens synthesis. Nascent collagens are hydroxylated in the ER through the activity of Prolyl-4-hydroxylases (P4h) and secreted in the ECM. A sudden increase of collagen synthesis/hydroxylation provokes a compartmentalized (ER->nucleus) metabolic perturbation of the substrates/cofactors (VitC and/or α-KG) of DNA/Histone hydroxylases (Tet, JMJ).

The aforementioned mechanisms allow cancer cells to survive and adjust to rapid, transient changes. Genetic mutations do not explain the heterogeneity/plasticity of cancer cells, which can be better explained by metabolic, epigenetic mechanisms. In this respect, we have recently proposed a novel mechanism underlying cancer cells plasticity by which collagen maturation may act as an epigenetic signal (57). Data provided in recently published papers underscore the existence of a functional link between Proline metabolism and epigenetic remodeling (84, 96, 97) and demonstrate that Proline availability influences mouse embryonic stem cell (mESC) identity and behavior through modulating AAR-ATF4 pathway (see above paragraph *Proline availability controls cancer cell behavior*). Of particular relevance is the finding that a sudden increase of Proline availability in mESCs induces a mesenchymal-like transition (esMT), which resembles the EMT that occurs at the invasive border of metastatic tumors (80, 84). This phenotypic transition is accompanied with metabolic and epigenetic changes similar to that observed in cancer cells. First, the acquisition of mesenchymal-like invasive features in Proline-treated ESCs (PiCs) is accompanied by a metabolic reprogramming shift from a bivalent to a glycolytic metabolism. Furthermore, esMT is accompanied by epigenetic remodeling, which results in a genome-wide increase of DNA and histone methylation (84, 98). While several metabolites act as cofactors or substrates of epigenetic enzymes and may thus influence the epigenetic landscape (99), a different mechanism has been recently proposed underlying Proline's epigenetic activity, which relies on collagen synthesis/maturation that requires Vitamin C (VitC) (Figure 2) (57). Following this model, consequently to a rapid increase of Proline-dependent collagen synthesis, the activity of Prolyl-hydroxylase (P4h) enzymes for collagen maturation, consumes VitC in the ER. This results in a reduced nuclear availability of VitC, which becomes limiting for the VitC/ αKG /Fe^+2^-dependent epigenetic enzymes, i.e., the JumonjiC-domain containing (JmjC) histone dioxygenases and the Ten-eleven Translocation (Tet) DNA demethylases, and determines a genome-wide increase of histones and DNA methylation (57, 98) (Figure 2). This functional interplay between P4H and JmjC/Tet enzymes explains, at least in part, the mechanism through which Proline induces the epigenetic remodeling associated with reversible esMT/MesT (57, 98). This previously unexplored functional interplay between Proline metabolism/collagen hydroxylation and epigenetic remodeling is not unique of ESCs but similarly occurs in cancer cells and contribute to breast cancer cell plasticity and metastatic progression (57) (Figure 2).

These findings lead us to hypothesize the existence of a Proline metabolism-dependent cycle of collagen synthesis and degradation in the same cell (Figure 2). Concomitant to continuous synthesis and maturation of collagen inside the cells, which promotes cancer cell invasiveness, collagen is degraded in the tumor microenvironment through the activity of the metalloproteinases (MMPs) and collagenases. This serves as reservoir of free extracellular Proline that in turn is taken up by the cell and used for protein synthesis/collagen biosynthesis, potentiating the cycle itself, and causing the epigenetic remodeling (Figure 2). Interestingly, genes encoding for MMP9, 1 and 13 and PREP, involved in proteins/collagen degradation are overexpressed in many different tumor types (26) (Table 1). Additional crucial enzymes required for collagen degradation and uptake may also contribute to the regeneration of this cycle, including prolidases (100), which hydrolyzes di- or tri-peptides with C-terminal Proline or hydroxyproline residues, and other collagenases.

In this context, interesting findings have been recently reported in pancreatic ductal adenocarcinoma (PDAC), which is characterized by cells organized in gland-like structures embedded in a dense collagen meshwork, which limits the delivery of nutrients and oxygen (101). Under nutrients starvation, PDAC cells can survive by using collagen-derived Proline as a source of energy. In fact, PDAC cells express Proline metabolic enzymes and are able to uptake collagen both through macropinocytosis (102) and uPARAP/Endo180 collagen receptor (101). Tracer experiments showed that upon collagen uptake, free Proline primarily contributes to biomass through incorporation into proteins, while only at small ratio in non-protein metabolic compartments. Genetic and pharmacological inhibition of PRODH, whose expression is increased in PDAC, significantly reduces PDAC cells clonogenicity *in vitro*, which is rescued by exogenous Proline, and strongly reduces pancreatic tumor growth *in vivo* (101). The authors suggest that collagen-derived Proline may promote cancer cell survival and proliferation through TCA cycle metabolism and cellular respiration; however, it would be interesting to investigate the fate of collagen-derived free Proline to protein synthesis as an additional/alternative mechanism of PDAC cells survival and invasiveness.

## Potential Therapeutic Strategies Targeting Proline Metabolism in Cancer

**Figure 3:**
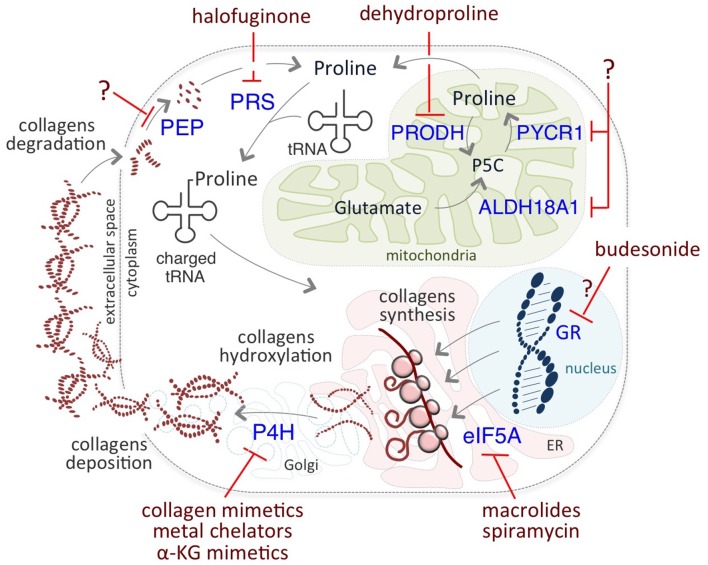
Putative therapeutic targets of Proline metabolism. Halofuginone (HF) binds to the glutamyl-prolyl-tRNA synthetase (PRS) and inhibits the prolyl-tRNA loading, reducing Proline-rich protein synthesis, such as collagens. Inhibitors of P4H enzymes include collagen mimetics, metal chelators, and α-KG mimetic. Macrolides, such as Spiramycin, inhibit the translation of mRNAs coding for Proline- rich stretches/motifs by blocking the elongation (eIF5A) of Proline- rich polypeptides. Budesonide reduces collagen synthesis and maturation, most likely acting as a GR antagonist. Dehydroproline (DHP) and L-tetrahydro-2-furoic acid (L-THFA) is a PRODH/POX inhibitor.

The critical role of Proline on cancer cell identity and behavior prompted researchers to develop novel therapeutic anti-cancer strategies targeting different levels of Proline metabolism (Figure 3). Among them, halofuginone (HF) holds great promise. HF is a derivative of febrifugine, a fundamental herb of traditional Chinese medicine, used to treat malaria for almost 2,000 years. HF binds the glutamyl-prolyl-tRNA synthetase (EPRS) and inhibits prolyl-tRNA formation (tRNA loading) (85, 86). This inhibition leads to the activation of the aminoacid stress response (AAR) pathway, which senses amino acid restriction through the accumulation of uncharged tRNAs, phosphorylation of Gcn2-eIF2α, and ATF4 expression (80). Increased levels of ATF4 activates the transcription of a set of genes that are crucial for the adaptation of cells to a stress environment (103). HF-dependent inhibition is reversed by exogenous supplementation of Proline.

HF has gained increasing interest for its potential application in the treatment of fibrosis and cancer since many studies have demonstrated its efficacy in inhibiting growth and progression of many types of tumors, mainly reducing collagen, and stroma accumulation, metalloproteinases, angiogenesis, and immune responses. In glioma tumors HF reduces collagen accumulation (104), in leiomyoma HF reduces cell proliferation *in vitro* (105) and *in vivo* (106); in bladder carcinoma HF inhibits angiogenesis, tumor stroma and growth (107). HF disrupts the stromal barriers also in PDAC, and modulates the immune response, leading to reduced tumor volume (108), and reduced subcutaneous pancreatic tumor development in a xenograft model (109). HF efficacy has been reported on prostate cancer growth *in vivo* (110) and on pheochromocytoma with vasculature reduction (111). Independent studies indicate that HF inhibits breast cancer growth by different mechanisms, including the induction of ROS production, which in turn activate apoptosis, and the inhibition of cell migration by down regulation of the matrix metalloproteinase 9 (MMP9) (112), or through activation of autophagy (113). HF reduces breast and prostate bone metastasis, by inhibiting TGFβ- and BMP- signaling (114). Recently, HF has been found to block breast cancer cells growth by controlling the exosome production of miRNAs involved in cell cycle and growth control (115). HF blocks colorectal cancer growth *in vitro* and *in vivo* through inhibition of AKT/mTORC1 pathway (116) and induction of autophagy under nutrient-rich conditions, and inhibition of autophagy under nutrient- poor conditions (117). HF suppresses HCC growth and progression (118, 119), lung metastasis by decreasing MMP activity (120). HF suppresses acute promyelocytic leukemia cell proliferation and promotes apoptosis (121), resulting in hematological remission *in vivo* (122); and inhibits multiple myeloma cell proliferation (123). HF augmented the sensitivity of different cancer cell lines to radiotherapy (124) and enhances the chemo-sensitivity of cancer cells (125), HF potentiates the radiotherapy effects of Lewis lung cancer cell *in vitro* and *in vivo* (126), reverting radiotherapy- dependent induction of TGFβ and EMT (127). HF is effective for the treatment of metastatic brain tumors (128), reduces melanoma bone metastasis, (129). Hence some pre- clinic models (130), and phase I and II clinical trials have been developed (131), showing the great potential of this molecule for cancer treatment.

The elongation factor P (EF-P, also known as eIF5A) is implicated in the translation of mRNAs coding for Proline- rich stretches/motifs (132, 133). Of note, eIF5A was associated to development of human cancers, and considered as an oncogene (47), and a potential therapeutic target (Table 1).

Macrolide antibiotics have been proposed as potential therapeutic targets relevant for inhibition of Proline-rich protein synthesis. Accordingly, it has been recently reported that macrolides can induce ribosome stalling, blocking the elongation of Proline- rich polypeptides (134). Interestingly, the macrolide Spiramycin is a potent inhibitor of Proline- induced esMT (57), suggesting that it may exert anti metastatic activities although it has not been yet tested in the cancer cell models.

Based on the well-known role of TGFβ in inducing collagens gene expression, other potential therapeutic targets that must be included in this section, as an extension of Proline metabolism targets for cancer treatment, are TGFβ inhibitors, which are the focus of many research projects and clinical trials (135).

The findings that collagen hydroxylation enzymes play a key role in cancer progression, and that their expression positively correlates with patient mortality, encouraged development of chemical inhibitors of P4H enzymes. These include metal ions and chelators, mimetics of the co-substrate α-KG, and collagen mimetic peptides. However, so far the majority of them have shown low clinical relevance, mainly because of high toxicity and/or low efficacy *in vivo* (136), suggesting that although promising, this field still needs further investigations.

In a very recent study, Budesonide, which is a drug commonly used to treat asthma and reduce collagen in pathological fibrotic conditions, has been identified as a candidate for the treatment of metastatic cancer (57). Indeed, Budesonide has been identified in a high-through-put phenotypic screening of 1200 FDA-approved drugs searching for esMT inhibitors. Budesonide not only antagonizes Proline-dependent esMT but also impairs the acquisition of mesenchymal and motile/invasive features in human lung and breast cancer cell lines, reducing collagen synthesis/accumulation *in vitro*. Furthermore, Budesonide impairs ECM and collagen accumulation in an orthotopic model of human breast cancer development *in vivo* and reduces metastasis formation (57). Although the mechanism of action of Budesonide is still far from fully elucidated, it may act as a glucocorticoid receptor (GR) antagonist. Interestingly, asthmatic patients under long-term treatment with Budesonide show reduced risk to develop pancreatic ductal adenocarcinoma (137). Moreover, Budesonide has been recently identified, using a combined connectivity mapping and pharmacoepidemiology approach, as a potential treatment for preventing breast cancer (138). Finally, by blocking collagen synthesis and hydroxylation in the ER, Budesonide increases VitC availability in the nucleus, resulting in epigenetic remodeling, i.e., a global reduction of DNA and histone methylation levels (57), thus showing its potential for targeting cancer cell plasticity and identity.

Finally, PYCR1 is considered a potential therapeutic target in HCC (27) and in BC (31) but so far, specific inhibitors of PYCR1 are not available.

## Conclusions and Perspectives

Proline is abundantly incorporated into collagen and provides the structural strength in higher animals. Besides this well-know function of Proline/collagen, increasing attention has been drawn to the impact of Proline and collagen metabolism, i.e., collagen synthesis/hydroxylation and degradation, on cell identity and behavior (14, 57). In this context, our recent findings that VitC-dependent collagen hydroxylation influences the epigenetic landscape and contributes to cellular plasticity (57) open new and important perspectives.

It has been proposed that oncogenic mutations may perturb the function of metabolic enzymes, which may in turn act as oncogenes. We suggest that such mutations, along with cancer cell metabolic reprogramming, may alter the availability of specific metabolites/cofactors required by epigenetic enzymes, e.g., Vitamin C, and alter the epigenetic signature of cancer cells causing, at least in part, tumor heterogeneity.

Following our proposed model, a functional interplay between Proline metabolism/collagen biosynthesis and epigenetic remodeling may generate a cycle based on the concomitant collagen synthesis and degradation, which sustains the cycle itself and controls cancer cell plasticity and behavior. Development of therapeutic strategies targeting this metabolic axis may provide novel options to target cancer cell heterogeneity.

A critical aspect is that proliferating cancer cells develop significant metabolic heterogeneity in order to survive to changing microenvironment (5). This heterogeneity is of particular relevance when envisioning therapeutic strategies and represents the main roadblock to attempts to control cell proliferation. The combination of cocktails of drugs that target metabolic pathways at different levels may represent a successful strategy to ultimately eradicate metastasizing cancer cells. In this context, several therapeutic strategies have been proposed that target Proline metabolism at different levels, including inhibitors at the level of prolyl-tRNA synthetase/collagen synthesis and collagen prolyl-hydroxylation. Although these options hold great promise, further investigations and clinical applications are needed to validate potential efficacy.

## Author Contributions

CD'A prepared the draft of the manuscript with input from all the authors. EP designed and prepared the figures. EP, JP, and GM revised the draft. All authors read, edited and approved the final manuscript.

## Conflict of Interest

The authors declare that the research was conducted in the absence of any commercial or financial relationships that could be construed as a potential conflict of interest.

## References

[1] Gupta PB Pastushenko I Skibinski A Blanpain C Kuperwasser C. Phenotypic plasticity: driver of cancer initiation, progression, and therapy resistance. Cell Stem Cell. (2019) 24:65–78. 10.1016/j.stem.2018.11.011 30554963 PMC7297507

[2] Saygin C Matei D Majeti R Reizes O Lathia JD. Targeting cancer stemness in the clinic: from hype to hope. Cell Stem Cell. (2019) 24:25–40. 10.1016/j.stem.2018.11.017 30595497

[3] Valastyan S Weinberg RA. Tumor metastasis: molecular insights and evolving paradigms. Cell. (2011) 147:275–92. 10.1016/j.cell.2011.09.024 22000009 PMC3261217

[4] Pastushenko I Brisebarre A Sifrim A Fioramonti M Revenco T Boumahdi S. Identification of the tumour transition states occurring during EMT. Nature. (2018) 556:463–8. 10.1038/s41586-018-0040-3 29670281

[5] Pavlova NN Thompson CB. The emerging hallmarks of cancer metabolism. Cell Metab. (2016) 23:27–47. 10.1016/j.cmet.2015.12.006 26771115 PMC4715268

[6] Geck RC Toker A. Nonessential amino acid metabolism in breast cancer. Adv Biol Regul. (2016) 62:11–17. 10.1016/j.jbior.2016.01.001 26838061

[7] Choi BH Coloff JL. The diverse functions of non-essential amino acids in cancer. Cancers. (2019) 11:675. 10.3390/cancers11050675 31096630 PMC6562791

[8] Liu W Hancock CN Fischer JW Harman M Phang JM. Proline biosynthesis augments tumor cell growth and aerobic glycolysis: involvement of pyridine nucleotides. Sci Rep. (2015) 5:17206. 10.1038/srep17206 26598224 PMC4657043

[9] Page-McCaw Ewald AJ Werb Z. Matrix metalloproteinases and the regulation of tissue remodelling. Nat Rev Mol Cell Biol. (2007) 8:221–33. 10.1038/nrm2125 17318226 PMC2760082

[10] Phang JM Liu W Hancock CN Fischer JW. Proline metabolism and cancer: emerging links to glutamine and collagen. Curr Opin Clin Nutr Metab Care. (2015) 18:71–7. 10.1097/MCO.0000000000000121 25474014 PMC4255759

[11] Phang JM Liu W. Proline metabolism and cancer. Front Biosci. (2012) 17:1835–45. 10.2741/4022 22201839 PMC7467630

[12] Phang JM. The regulatory functions of proline and pyrroline-5-carboxylic acid. Curr Top Cell Regul. (1985) 25:91–132. 10.1016/B978-0-12-152825-6.50008-4 2410198

[13] Huynh TYL Zareba I Baszanowska W Lewoniewska S Palka J. Understanding the role of key amino acids in regulation of proline dehydrogenase/proline oxidase. (prodh/pox)-dependent apoptosis/autophagy as an approach to targeted cancer therapy. Mol Cell Biochem. (2020) 466:35–44. 10.1007/s11010-020-03685-y 31933109 PMC7028810

[14] Phang JM. Proline metabolism in cell regulation and cancer biology: recent advances and hypotheses. Antioxid Redox Signal. (2019) 30:635–49. 10.1089/ars.2017.7350 28990419 PMC6338564

[15] Maxwell SA Davis GE. Differential gene expression in p53-mediated apoptosis-resistant vs. apoptosis-sensitive tumor cell lines. Proc Natl Acad Sci USA. (2000) 97:13009–14. 10.1073/pnas.230445997 11069295 PMC27169

[16] Maxwell SA Rivera A. Proline oxidase induces apoptosis in tumor cells, and its expression is frequently absent or reduced in renal carcinomas. J Biol Chem. (2003) 278:9784–9. 10.1074/jbc.M210012200 12514185

[17] Donald SP Sun XY Hu CA Yu J Mei JM Valle D Phang JM. Proline oxidase, encoded by p53-induced gene-6, catalyzes the generation of proline-dependent reactive oxygen species. Cancer Res. (2001) 61:1810–5. 11280728

[18] Polyak K Xia Y Zweier JL Kinzler KW Vogelstein B. A model for p53-induced apoptosis. Nature. (1997) 389:300–5. 10.1038/38525 9305847

[19] Liu W Zabirnyk O Wang H Shiao YH Nickerson ML Khalil S. miR-23b targets proline oxidase, a novel tumor suppressor protein in renal cancer. Oncogene. (2010) 29:4914–24. 10.1038/onc.2010.237 20562915 PMC4398970

[20] Phang JM Liu W Zabirnyk O. Proline metabolism and microenvironmental stress. Annu Rev Nutr. (2010) 30:441–63. 10.1146/annurev.nutr.012809.104638 20415579 PMC7365493

[21] Tang L Zeng J Geng P Fang C Wang Y Sun M. Global metabolic profiling identifies a pivotal role of proline and hydroxyproline metabolism in supporting hypoxic response in hepatocellular carcinoma. Clin Cancer Res. (2018) 24:474–85. 10.1158/1078-0432.CCR-17-1707 29084919

[22] Liu Y Borchert GL Donald SP Diwan BA Anver M Phang JM. Proline oxidase functions as a mitochondrial tumor suppressor in human cancers. Cancer Res. (2009) 69:6414–22. 10.1158/0008-5472.CAN-09-1223 19654292 PMC4287397

[23] Liu W Phang JM. Proline dehydrogenase. (oxidase), a mitochondrial tumor suppressor, and autophagy under the hypoxia microenvironment. Autophagy. (2012) 8:1407–9. 10.4161/auto.21152 22885468 PMC3442893

[24] Liu W Phang JM. Proline dehydrogenase. (oxidase) in cancer. Biofactors. (2012) 38:398–406. 10.1002/biof.1036 22886911 PMC7479541

[25] Elia I Broekaert D Christen S Boon R Radaelli E Orth MF. Proline metabolism supports metastasis formation and could be inhibited to selectively target metastasizing cancer cells. Nat Commun. (2017) 8:15267. 10.1038/ncomms15267 28492237 PMC5437289

[26] Nilsson R Jain M Madhusudhan N Sheppard NG Strittmatter L Kampf C. Metabolic enzyme expression highlights a key role for MTHFD2 and the mitochondrial folate pathway in cancer. Nat Commun. (2014) 5:3128. 10.1038/ncomms4128 24451681 PMC4106362

[27] Ding Z Ericksen RE Escande-Beillard N Lee QY Loh A Denil S. Metabolic pathway analyses identify proline biosynthesis pathway as a promoter of liver tumorigenesis. J Hepatol. (2019) 72:725–35. 10.1016/j.jhep.2019.10.026 31726117

[28] Craze ML Cheung H Jewa N Coimbra NDM Soria D El-Ansari R. MYC regulation of glutamine-proline regulatory axis is key in luminal B breast cancer. Br J Cancer. (2018) 118:258–65. 10.1038/bjc.2017.387 29169183 PMC5785743

[29] Liu M Wang Y Yang C Ruan Y Bai C Chu Q. Inhibiting both proline biosynthesis and lipogenesis synergistically suppresses tumor growth. J Exp Med. (2020) 217:e20191226. 10.1084/jem.20191226 31961917 PMC7062513

[30] Zhuang J Song Y Ye Y He S Ma X Zhang M. PYCR1 interference inhibits cell growth and survival via c-Jun N-terminal kinase/insulin receptor substrate 1. (JNK/IRS1) pathway in hepatocellular cancer. J Transl Med. (2019) 17:343. 10.1186/s12967-019-2091-0 31619254 PMC6796468

[31] Ding J Kuo ML Su L Xue L Luh F Zhang H. Human mitochondrial pyrroline-5-carboxylate reductase 1 promotes invasiveness and impacts survival in breast cancers. Carcinogenesis. (2017) 38:519–31. 10.1093/carcin/bgx022 28379297

[32] Choi UY Lee JJ Park A Zhu W Lee HR Choi YJ. Oncogenic human herpesvirus hijacks proline metabolism for tumorigenesis. Proc Natl Acad Sci USA. (2020) 117:8083–93. 10.1073/pnas.1918607117 32213586 PMC7149499

[33] Zeng T Zhu L Liao M Zhuo W Yang S Wu W. Knockdown of PYCR1 inhibits cell proliferation and colony formation via cell cycle arrest and apoptosis in prostate cancer. Med Oncol. (2017) 34:27. 10.1007/s12032-016-0870-5 28078560

[34] Weijin F Zhibin X Shengfeng Z Xiaoli Y Qijian D Jiayi L. The clinical significance of PYCR1 expression in renal cell carcinoma. Medicine. (2019) 98:e16384. 10.1097/MD.0000000000016384 31305441 PMC6641676

[35] Wang QL Liu L. PYCR1 is associated with papillary renal cell carcinoma progression. Open Med. (2019) 14:586–92. 10.1515/med-2019-0066 31428683 PMC6698050

[36] Ye Y Wu Y Wang J. Pyrroline-5-carboxylate reductase 1 promotes cell proliferation via inhibiting apoptosis in human malignant melanoma. Cancer Manag Res. (2018) 10:6399–407. 10.2147/CMAR.S166711 30568501 PMC6267761

[37] Cai F Miao Y Liu C Wu T Shen S Su X. Pyrroline-5-carboxylate reductase 1 promotes proliferation and inhibits apoptosis in non-small cell lung cancer. Oncol Lett. (2018) 15:731–40. 10.3892/ol.2017.7400 29403556 PMC5780737

[38] Wang D Wang L Zhang Y Yan Z Liu L. PYCR1 promotes the progression of non-small-cell lung cancer under the negative regulation of miR-488. Biomed Pharmacother. (2019) 111:588–95. 10.1016/j.biopha.2018.12.089 30605882

[39] Sang S Zhang C Shan J. Pyrroline-5-carboxylate reductase 1 accelerates the migration and invasion of nonsmall cell lung cancer *in vitro*. Cancer Biother Radiopharm. (2019) 34:380–7. 10.1089/cbr.2019.2782 30916574

[40] She Y Mao A Li F Wei X. P5CR1 protein expression and the effect of gene-silencing on lung adenocarcinoma. PeerJ. (2019) 7:e6934. 10.7717/peerj.6934 31143549 PMC6524628

[41] K.Hollinshead ER Munford H Eales1 KL Bardella C Li C Escribano-Gonzalez C. Oncogenic IDH1 mutations promote enhanced proline synthesis through PYCR1 to support the maintenance of mitochondrial redox homeostasis. Cell Rep. (2018) 22:3107–14. 10.1016/j.celrep.2018.02.084 29562167 PMC5883319

[42] Fang E Wang X Yang F Hu A Wang J Li D. Therapeutic Targeting of MZF1-AS1/PARP1/E2F1 axis inhibits proline synthesis and neuroblastoma progression. Adv Sci. (2019) 6:1900581. 10.1002/advs.201900581 31592410 PMC6774027

[43] Sun T Song Y Yu H Luo X. Identification of lncRNA TRPM2-AS/miR-140–3p/PYCR1 axis's proliferates and anti-apoptotic effect on breast cancer using co-expression network analysis. Cancer Biol Ther. (2019) 20:760–73. 10.1080/15384047.2018.1564563 30810442 PMC6605980

[44] Chen S Yang X Yu M Wang Z Liu B Liu M. SIRT3 regulates cancer cell proliferation through deacetylation of PYCR1 in proline metabolism. Neoplasia. (2019) 21:665–75. 10.1016/j.neo.2019.04.008 31108370 PMC6526305

[45] Fang H Du G Wu Q Liu R Chen C Feng J. HDAC inhibitors induce proline dehydrogenase. (POX) transcription and anti-apoptotic autophagy in triple negative breast cancer. Acta Biochim Biophys Sin. (Shanghai). (2019) 51:1064–70. 10.1093/abbs/gmz097 31559416

[46] Liu Y Mao C Wang M Liu N Ouyang L Liu S. Cancer progression is mediated by proline catabolism in non-small cell lung cancer. Oncogene. (2020) 39:2358–76. 10.1038/s41388-019-1151-5 31911619

[47] Mathews MB Hershey JW. The translation factor eIF5A and human cancer. Biochim Biophys Acta. (2015) 1849:836–44. 10.1016/j.bbagrm.2015.05.002 25979826 PMC4732523

[48] Gilkes DM Chaturvedi P Bajpai S Wong CC Wei H Pitcairn S Hubbi ME. Collagen prolyl hydroxylases are essential for breast cancer metastasis. Cancer Res. (2013) 73:3285–96. 10.1158/0008-5472.CAN-12-3963 23539444 PMC3674184

[49] Xiong G Stewart RL Chen J Gao T Scott TL Samayoa LM. Collagen prolyl 4-hydroxylase 1 is essential for HIF-1alpha stabilization and TNBC chemoresistance. Nat Commun. (2018) 9:4456. 10.1038/s41467-018-06893-9 30367042 PMC6203834

[50] Cao XP Cao Y Li WJ Zhang HH Zhu ZM. P4HA1/HIF1alpha feedback loop drives the glycolytic and malignant phenotypes of pancreatic cancer. Biochem Biophys Res Commun. (2019) 516:606–12. 10.1016/j.bbrc.2019.06.096 31239153

[51] Li Q Shen Z Wu Z Shen Y Deng H Zhou C. High P4HA1 expression is an independent prognostic factor for poor overall survival and recurrent-free survival in head and neck squamous cell carcinoma. J Clin Lab Anal. (2019) e23107. 10.1002/jcla.23107 31782831 PMC7083458

[52] Kappler M Kotrba J Kaune T Bache M Rot S Bethmann D. P4HA1: a single-gene surrogate of hypoxia signatures in oral squamous cell carcinoma patients. Clin Transl Radiat Oncol. (2017) 5:6–11. 10.1016/j.ctro.2017.05.002 29594211 PMC5833914

[53] Chakravarthi BV Pathi SS Goswami MT Cieslik M Zheng H Nallasivam S. The miR-124-prolyl hydroxylase P4HA1-MMP1 axis plays a critical role in prostate cancer progression. Oncotarget. (2014) 5:6654–69. 10.18632/oncotarget.2208 25115393 PMC4196154

[54] Duan Y Dong Y Dang R Hu Z Yang Y Hu Y. MiR-122 inhibits epithelial mesenchymal transition by regulating P4HA1 in ovarian cancer cells. Cell Biol Int. (2018) 42:1564–74. 10.1002/cbin.11052 30136751

[55] Zhou Y Jin G Mi R Zhang J Zhang J Xu H. Knockdown of P4HA1 inhibits neovascularization via targeting glioma stem cell-endothelial cell transdifferentiation and disrupting vascular basement membrane. Oncotarget. (2017) 8:35877–89. 10.18632/oncotarget.16270 28415787 PMC5482624

[56] Hu WM Zhang J Sun SX Xi SY Chen ZJ Jiang XB. Identification of P4HA1 as a prognostic biomarker for high-grade gliomas. Pathol Res Pract. (2017) 213:1365–9. 10.1016/j.prp.2017.09.017 28964577

[57] D'Aniello C Cermola F Palamidessi A Wanderlingh LG Gagliardi M Migliaccio A. Collagen prolyl hydroxylation-dependent metabolic perturbation governs epigenetic remodeling and mesenchymal transition in pluripotent and cancer cells. Cancer Res. (2019) 79:3235–50. 10.1158/0008-5472.CAN-18-2070 31061065

[58] Lin J Wu J Wei X Gao C Jin Z Cui Y P4HA2, a prognostic factor, promotes glioma proliferation, invasion, migration and EMT through collagen regulation and PI3K/AKT pathway. bioRxiv [preprint] (2020). 10.1101/2020.02.05.935221

[59] Li Q Wang Q Zhang Q Zhang J Zhang J. Collagen prolyl 4-hydroxylase 2 predicts worse prognosis and promotes glycolysis in cervical cancer. Am J Transl Res. (2019) 11:6938–51. 31814898 PMC6895525

[60] Jarzab B Wiench M Fujarewicz K Simek K Jarzab M Oczko-Wojciechowska M. Gene expression profile of papillary thyroid cancer: sources of variability and diagnostic implications. Cancer Res. (2005) 65:1587–97. 10.1158/0008-5472.CAN-04-3078 15735049

[61] Jiang W Zhou X Li Z Liu K Wang W Tan R. Prolyl 4-hydroxylase 2 promotes B-cell lymphoma progression via hydroxylation of Carabin. Blood. (2018) 131:1325–36. 10.1182/blood-2017-07-794875 29437589 PMC5865229

[62] Chang KP Yu JS Chien KY Lee CW Liang Y Liao CT. Identification of PRDX4 and P4HA2 as metastasis-associated proteins in oral cavity squamous cell carcinoma by comparative tissue proteomics of microdissected specimens using iTRAQ technology. J Proteome Res. (2011) 10:4935–47. 10.1021/pr200311p 21859152

[63] Reis PP Waldron L Perez-Ordonez B Pintilie M Galloni NN Xuan Y. A gene signature in histologically normal surgical margins is predictive of oral carcinoma recurrence. BMC Cancer. (2011) 11:437. 10.1186/1471-2407-11-437 21989116 PMC3198722

[64] Feng GX Li J Yang Z Zhang SQ Liu YX Zhang WY. Hepatitis B virus X protein promotes the development of liver fibrosis and hepatoma through downregulation of miR-30e targeting P4HA2 mRNA. Oncogene. (2017) 36:6895–905. 10.1038/onc.2017.291 28846110

[65] Wang T Fu X Jin T Zhang L Liu B Wu Y. Aspirin targets P4HA2 through inhibiting NF-kappaB and LMCD1-AS1/let-7g to inhibit tumour growth and collagen deposition in hepatocellular carcinoma. EBioMedicine. (2019) 45:168–80. 10.1016/j.ebiom.2019.06.048 31278071 PMC6642319

[66] Xiong G Deng L Zhu J Rychahou PG Xu R. Prolyl-4-hydroxylase alpha subunit 2 promotes breast cancer progression and metastasis by regulating collagen deposition. BMC Cancer. (2014) 14:1. 10.1186/1471-2407-14-1 24383403 PMC3880410

[67] Toss MS Miligy IM Gorringe KL AlKawaz A Khout H Ellis IO. Prolyl-4-hydroxylase Alpha subunit 2. (P4HA2) expression is a predictor of poor outcome in breast ductal carcinoma in situ. (DCIS). Br J Cancer. (2018) 119:1518–26. 10.1038/s41416-018-0337-x 30410060 PMC6288166

[68] Xia W Zhuang J Wang G Ni J Wang J Ye Y. P4HB promotes HCC tumorigenesis through downregulation of GRP78 and subsequent upregulation of epithelial-to-mesenchymal transition. Oncotarget. (2017) 8:8512–21. 10.18632/oncotarget.14337 28052026 PMC5352418

[69] Wang SM Lin LZ Zhou DH Zhou JX Xiong SQ. Expression of prolyl 4-hydroxylase beta-polypeptide in non-small cell lung cancer treated with Chinese medicines. Chin J Integr Med. (2015) 21:689–96. 10.1007/s11655-013-1535-2 24382781

[70] Zhang J Wu Y Lin YH Guo S Ning PF Zheng ZC. Prognostic value of hypoxia-inducible factor-1 alpha and prolyl 4-hydroxylase beta polypeptide overexpression in gastric cancer. World J Gastroenterol. (2018) 24:2381–91. 10.3748/wjg.v24.i22.2381 29904245 PMC6000295

[71] Sun S Wong TS Zhang XQ Pu JK Lee NP Day PJ. Protein alterations associated with temozolomide resistance in subclones of human glioblastoma cell lines. J Neurooncol. (2012) 107:89–100. 10.1007/s11060-011-0729-8 21979894 PMC3273683

[72] Zhang J Guo S Wu Y Zheng ZC Wang Y Zhao Y. P4HB, a novel hypoxia target gene related to gastric cancer invasion and metastasis. Biomed Res Int. (2019) 2019:9749751. 10.1155/2019/9749751 31467922 PMC6699373

[73] I.Wortel MN van der Meer LT Kilberg MS van Leeuwen FN. Surviving stress: modulation of ATF4-mediated stress responses in normal and malignant cells. Trends Endocrinol Metab. (2017) 28:794–806. 10.1016/j.tem.2017.07.003 28797581 PMC5951684

[74] Singleton DC Harris AL. Targeting the ATF4 pathway in cancer therapy. Expert Opin Ther Targets. (2012) 16:1189–202. 10.1517/14728222.2012.728207 23009153

[75] Tanner JJ Fendt SM Becker DF. The proline cycle as a potential cancer therapy target. Biochemistry. (2018) 57:3433–44. 10.1021/acs.biochem.8b00215 29648801 PMC6026536

[76] De Ingeniis J Ratnikov B Richardson AD Scott DA Aza-Blanc P De SK. Functional specialization in proline biosynthesis of melanoma. PLoS ONE. (2012) 7:e45190. 10.1371/journal.pone.0045190 23024808 PMC3443215

[77] Liu W Le A Hancock C Lane AN Dang CV Fan TW. Reprogramming of proline and glutamine metabolism contributes to the proliferative and metabolic responses regulated by oncogenic transcription factor c-MYC. Proc Natl Acad Sci USA. (2012) 109:8983–8. 10.1073/pnas.1203244109 22615405 PMC3384197

[78] Loayza-Puch F Rooijers K Buil LC Zijlstra J Oude Vrielink JF Lopes R. Tumour-specific proline vulnerability uncovered by differential ribosome codon reading. Nature. (2016) 530:490–4. 10.1038/nature16982 26878238

[79] Sahu N Dela Cruz D Gao M Sandoval W Haverty PM Liu J. Proline starvation induces unresolved ER stress and hinders mTORC1-dependent tumorigenesis. Cell Metab. (2016) 24:753–61. 10.1016/j.cmet.2016.08.008 27618686

[80] D'Aniello C Fico A Casalino L Guardiola O Di Napoli G Cermola F. A novel autoregulatory loop between the Gcn2-Atf4 pathway and. (L)-Proline [corrected] metabolism controls stem cell identity. Cell Death Differ. (2015) 22:1094–5. 10.1038/cdd.2015.24 25857264 PMC4572871

[81] Tan BS Lonic A Morris MB Rathjen PD Rathjen J The amino acid transporter SNAT2 mediates L-proline-induced differentiation of ES cells. American journal of physiology. Cell Physiol. (2011) 300:C1270–9. 10.1152/ajpcell.00235.2010 21346154

[82] Han J Back SH Hur J Lin YH Gildersleeve R Shan J. ER-stress-induced transcriptional regulation increases protein synthesis leading to cell death. Nature Cell Biol. (2013) 15:481–90. 10.1038/ncb2738 23624402 PMC3692270

[83] Palii SS Chen H Kilberg MS. Transcriptional control of the human sodium-coupled neutral amino acid transporter system A gene by amino acid availability is mediated by an intronic element. J Biol Chem. (2004) 279:3463–71. 10.1074/jbc.M310483200 14623874

[84] Comes S Gagliardi M Laprano N Fico A Cimmino A Palamidessi A. L-Proline induces a mesenchymal-like invasive program in embryonic stem cells by remodeling H3K9 and H3K36 methylation. Stem Cell Reports. (2013) 1:307–21. 10.1016/j.stemcr.2013.09.001 24319666 PMC3849245

[85] Keller TL Zocco D Sundrud MS Hendrick M Edenius M Yum J. Halofuginone and other febrifugine derivatives inhibit prolyl-tRNA synthetase. Nat Chem Biol. (2012) 8:311–7. 10.1038/nchembio.790 22327401 PMC3281520

[86] Zhou H Sun L Yang XL Schimmel P. ATP-directed capture of bioactive herbal-based medicine on human tRNA synthetase. Nature. (2013) 494:121–4. 10.1038/nature11774 23263184 PMC3569068

[87] Morrison SJ Spradling AC. Stem cells and niches: mechanisms that promote stem cell maintenance throughout life. Cell. (2008) 132:598–611. 10.1016/j.cell.2008.01.038 18295578 PMC4505728

[88] Scadden DT. Nice neighborhood: emerging concepts of the stem cell niche. Cell. (2014) 157:41–50. 10.1016/j.cell.2014.02.013 24679525 PMC4161226

[89] Lane SW Williams DA Watt FM. Modulating the stem cell niche for tissue regeneration. Nat Biotechnol. (2014) 32:795–803. 10.1038/nbt.2978 25093887 PMC4422171

[90] Myllyharju J. Prolyl 4-hydroxylases, the key enzymes of collagen biosynthesis. Matrix Biol. (2003) 22:15–24. 10.1016/S0945-053X(03)00006-4 12714038

[91] Bordoli MR Yum J Breitkopf SB Thon JN Italiano JE Jr Xiao J. A secreted tyrosine kinase acts in the extracellular environment. Cell. (2014) 158:1033–44. 10.1016/j.cell.2014.06.048 25171405 PMC4149754

[92] Schworer S Berisa M Violante S Qin W Zhu J Hendrickson RC Proline biosynthesis is a vent for TGFbeta-induced mitochondrial redox stress. EMBO J. (2020) 5:e103334 10.15252/embj.2019103334 PMC7156964 32134147

[93] Xu S Xu H Wang W Li S Li H Li T. The role of collagen in cancer: from bench to bedside. J Transl Med. (2019) 17:309. 10.1186/s12967-019-2058-1 31521169 PMC6744664

[94] Pankova D Jiang Y Chatzifrangkeskou M Vendrell I Buzzelli J Ryan A. RASSF1A controls tissue stiffness and cancer stem-like cells in lung adenocarcinoma. EMBO J. (2019) 38:e100532. 10.15252/embj.2018100532 31268606 PMC6600643

[95] Guo L Cui C Zhang K Wang J Wang Y Lu Y. Kindlin-2 links mechano-environment to proline synthesis and tumor growth. Nat Commun. (2019) 10:845. 10.1038/s41467-019-08772-3 30783087 PMC6381112

[96] Casalino L Comes S Lambazzi G De Stefano B Filosa S De Falco S. Control of embryonic stem cell metastability by L-proline catabolism. J Mol Cell Biol. (2011) 3:108–22. 10.1093/jmcb/mjr001 21307025

[97] D'Aniello C Cermola F Patriarca EJ Minchiotti G. Vitamin C in stem cell biology: impact on extracellular matrix homeostasis and epigenetics. Stem Cells Int. (2017) 2017:8936156. 10.1155/2017/8936156 28512473 PMC5415867

[98] D'Aniello C Habibi E Cermola F Paris D Russo F Fiorenzano A. Vitamin C and l-proline antagonistic effects capture alternative states in the pluripotency continuum. Stem Cell Reports. (2017) 8:11. 10.1016/j.stemcr.2016.11.011 28017658 PMC5233408

[99] D'Aniello C Cermola F Patriarca EJ Minchiotti G Metabolic–epigenetic axis in pluripotent state transitions. Epigenomes. (2019) 3:13 10.3390/epigenomes3030013 PMC8594706 34968225

[100] Surazynski A Miltyk W Palka J Phang JM. Prolidase-dependent regulation of collagen biosynthesis. Amino Acids. (2008) 35:731–8. 10.1007/s00726-008-0051-8 18320291

[101] Olivares O Mayers JR Gouirand V Torrence ME Gicquel T Borge L. Collagen-derived proline promotes pancreatic ductal adenocarcinoma cell survival under nutrient limited conditions. Nat Commun. (2017) 8:16031. 10.1038/ncomms16031 28685754 PMC5504351

[102] Kerr MC Teasdale RD. Defining macropinocytosis. Traffic. (2009) 10:364–71. 10.1111/j.1600-0854.2009.00878.x 19192253

[103] Kilberg MS Pan YX Chen H Leung-Pineda V. Nutritional control of gene expression: how mammalian cells respond to amino acid limitation. Annu Rev Nutr. (2005) 25:59–85. 10.1146/annurev.nutr.24.012003.132145 16011459 PMC3600373

[104] Abramovitch R Dafni H Neeman M Nagler A Pines M. Inhibition of neovascularization and tumor growth, and facilitation of wound repair, by halofuginone, an inhibitor of collagen type I synthesis. Neoplasia. (1999) 1:321–9. 10.1038/sj.neo.7900043 10935487 PMC1508102

[105] Grudzien MM Low PS Manning PC Arredondo M Belton RJ Jr Nowak RA. The antifibrotic drug halofuginone inhibits proliferation and collagen production by human leiomyoma and myometrial smooth muscle cells. Fertil Steril. (2010) 93:1290–8. 10.1016/j.fertnstert.2008.11.018 19135664 PMC2860739

[106] Koohestani F Qiang W MacNeill AL Druschitz SA Serna VA Adur M. Halofuginone suppresses growth of human uterine leiomyoma cells in a mouse xenograft model. Hum Reprod. (2016) 31:1540–51. 10.1093/humrep/dew094 27130615 PMC4901881

[107] Elkin M Ariel I Miao HQ Nagler A Pines M de-Groot N. Inhibition of bladder carcinoma angiogenesis, stromal support, and tumor growth by halofuginone. Cancer Res. (1999) 59:4111–8. 10463616

[108] Elahi-Gedwillo KY Carlson M Zettervall J Provenzano PP. Antifibrotic therapy disrupts stromal barriers and modulates the immune landscape in pancreatic ductal adenocarcinoma. Cancer Res. (2019) 79:372–86. 10.1158/0008-5472.CAN-18-1334 30401713 PMC6335156

[109] Spector I Honig H Kawada N Nagler A Genin O Pines M. Inhibition of pancreatic stellate cell activation by halofuginone prevents pancreatic xenograft tumor development. Pancreas. (2010) 39:1008–15. 10.1097/MPA.0b013e3181da8aa3 20442678

[110] Gavish Z Pinthus JH Barak V Ramon J Nagler A Eshhar Z. Growth inhibition of prostate cancer xenografts by halofuginone. Prostate. (2002) 51:73–83. 10.1002/pros.10059 11948962

[111] Gross DJ Reibstein I Weiss L Slavin S Dafni H Neeman M. Treatment with halofuginone results in marked growth inhibition of a von Hippel-Lindau pheochromocytoma *in vivo*. Clin Cancer Res. (2003) 9:3788–93. 14506172

[112] Jin ML Park SY Kim YH Park G Lee SJ. Halofuginone induces the apoptosis of breast cancer cells and inhibits migration via downregulation of matrix metalloproteinase-9. Int J Oncol. (2014) 44:309–18. 10.3892/ijo.2013.2157 24173318

[113] Xia X Wang L Zhang X Wang S Lei L Cheng L. Halofuginone-induced autophagy suppresses the migration and invasion of MCF-7 cells via regulation of STMN1 and p53. J Cell Biochem. (2018) 119:4009–20. 10.1002/jcb.26559 29231257

[114] Juarez P P.Fournier GJ Mohammad KS McKenna RC Davis HW Peng XH. Halofuginone inhibits TGF-beta/BMP signaling and in combination with zoledronic acid enhances inhibition of breast cancer bone metastasis. Oncotarget. (2017) 8:86447–62. 10.18632/oncotarget.21200 29156807 PMC5689697

[115] Xia X Wang X Zhang S Zheng Y Wang L Xu Y. miR-31 shuttled by halofuginone-induced exosomes suppresses MFC-7 cell proliferation by modulating the HDAC2/cell cycle signaling axis. J Cell Physiol. (2019) 234:18970–84. 10.1002/jcp.28537 30916359

[116] Chen GQ Tang CF Shi XK Lin CY Fatima S Pan XH. Halofuginone inhibits colorectal cancer growth through suppression of Akt/mTORC1 signaling and glucose metabolism. Oncotarget. (2015) 6:24148–62. 10.18632/oncotarget.4376 26160839 PMC4695176

[117] Chen GQ Gong RH Yang DJ Zhang G Lu AP Yan SC. Halofuginone dually regulates autophagic flux through nutrient-sensing pathways in colorectal cancer. Cell Death Dis. (2017) 8:e2789. 10.1038/cddis.2017.203 28492544 PMC5520722

[118] Nagler A Ohana M Shibolet O Shapira MY Alper R Vlodavsky I. Suppression of hepatocellular carcinoma growth in mice by the alkaloid coccidiostat halofuginone. Eur J Cancer. (2004) 40:1397–403. 10.1016/j.ejca.2003.11.036 15177499

[119] Huo S Yu H Li C Zhang J Liu T. Effect of halofuginone on the inhibition of proliferation and invasion of hepatocellular carcinoma HepG2 cell line. Int J Clin Exp Pathol. (2015) 8:15863–70. 26884857 PMC4730070

[120] Taras D Blanc JF Rullier A Dugot-Senant N Laurendeau I Bieche I. Halofuginone suppresses the lung metastasis of chemically induced hepatocellular carcinoma in rats through MMP inhibition. Neoplasia. (2006) 8:312–8. 10.1593/neo.05796 16756723 PMC1600678

[121] de Figueiredo-Pontes LL Assis PA Santana-Lemos BA Jacomo RH Lima AS Garcia AB. Halofuginone has anti-proliferative effects in acute promyelocytic leukemia by modulating the transforming growth factor beta signaling pathway. PLoS ONE. (2011) 6:e26713. 10.1371/journal.pone.0026713 22053203 PMC3203897

[122] Assis PA De Figueiredo-Pontes LL Lima AS Leao V Candido LA Pintao CT. Halofuginone inhibits phosphorylation of SMAD-2 reducing angiogenesis and leukemia burden in an acute promyelocytic leukemia mouse model. J Exp Clin Cancer Res. (2015) 34:65. 10.1186/s13046-015-0181-2 26099922 PMC4486128

[123] Leiba M Jakubikova J Klippel S Mitsiades CS Hideshima T Tai YT. Halofuginone inhibits multiple myeloma growth in vitro and in vivo and enhances cytotoxicity of conventional and novel agents. Br J Haematol. (2012) 157:718–31. 10.1111/j.1365-2141.2012.09120.x 22533681 PMC4414398

[124] Cook JA Choudhuri R Degraff W Gamson J Mitchell JB. Halofuginone enhances the radiation sensitivity of human tumor cell lines. Cancer Lett. (2010) 289:119–26. 10.1016/j.canlet.2009.08.009 19713035 PMC2835928

[125] Tsuchida K Tsujita T Hayashi M Ojima A Keleku-Lukwete N Katsuoka F Otsuki A. Halofuginone enhances the chemo-sensitivity of cancer cells by suppressing NRF2 accumulation. Free Radic Biol Med. (2017) 103:236–47. 10.1016/j.freeradbiomed.2016.12.041 28039084

[126] Lin R Yi S Gong L Liu W Wang P Liu N. Inhibition of TGF-beta signaling with halofuginone can enhance the antitumor effect of irradiation in Lewis lung cancer. Onco Targets Ther. (2015) 8:3549–59. 10.2147/OTT.S92518 26664138 PMC4671802

[127] Chen Y Liu W Wang P Hou H Liu N Gong L. Halofuginone inhibits radiotherapy-induced epithelial-mesenchymal transition in lung cancer. Oncotarget. (2016) 7:71341–52. 10.18632/oncotarget.11217 27533085 PMC5342082

[128] Abramovitch R Itzik A Harel H Nagler A Vlodavsky I Siegal T. Halofuginone inhibits angiogenesis and growth in implanted metastatic rat brain tumor model–an MRI study. Neoplasia. (2004) 6:480–9. 10.1593/neo.03520 15548356 PMC1635242

[129] Juarez P Mohammad KS Yin JJ Fournier PG McKenna RC Davis HW. Halofuginone inhibits the establishment and progression of melanoma bone metastases. Cancer Res. (2012) 72:6247–56. 10.1158/0008-5472.CAN-12-1444 23002206 PMC4447239

[130] Lamora A Mullard M Amiaud J Brion R Heymann D Redini F. Anticancer activity of halofuginone in a preclinical model of osteosarcoma: inhibition of tumor growth and lung metastases. Oncotarget. (2015) 6:14413–27. 10.18632/oncotarget.3891 26015407 PMC4546476

[131] de Jonge MJ Dumez H Verweij J Yarkoni S Snyder D Lacombe D. Phase I and pharmacokinetic study of halofuginone, an oral quinazolinone derivative in patients with advanced solid tumours. Eur J Cancer. (2006) 42:1768–74. 10.1016/j.ejca.2005.12.027 16815702

[132] Gutierrez E Shin BS Woolstenhulme CJ Kim JR Saini P Buskirk AR. eIF5A promotes translation of polyproline motifs. Mol Cell. (2013) 51:35–45. 10.1016/j.molcel.2013.04.021 23727016 PMC3744875

[133] Doerfel LK Wohlgemuth I Kothe C Peske F Urlaub H Rodnina MV. EF-P is essential for rapid synthesis of proteins containing consecutive proline residues. Science. (2013) 339:85–8. 10.1126/science.1229017 23239624

[134] Davis AR Gohara DW Yap MN. Sequence selectivity of macrolide-induced translational attenuation. Proc Natl Acad Sci USA. (2014) 111:15379–84. 10.1073/pnas.1410356111 25313041 PMC4217412

[135] Lahn M Kloeker S Berry BS. TGF-beta inhibitors for the treatment of cancer. Exp Opin Investig Drugs. (2005) 14:629–43. 10.1517/13543784.14.6.629 16004592

[136] Vasta JD Raines RT. Collagen Prolyl 4-hydroxylase as a therapeutic target. J Med Chem. (2018) 61:10403–411. 10.1021/acs.jmedchem.8b00822 29986141 PMC6344319

[137] Gomez-Rubio P Zock JP Rava M Marquez M Sharp L Hidalgo M. Reduced risk of pancreatic cancer associated with asthma and nasal allergies. Gut. (2017) 66:314–22. 10.1136/gutjnl-2015-310442 26628509

[138] Busby J Murray L Mills K Zhang SD Liberante F Cardwell CR. A combined connectivity mapping and pharmacoepidemiology approach to identify existing medications with breast cancer causing or preventing properties. Pharmacoepidemiol Drug Saf. (2018) 27:78–86. 10.1002/pds.4345 29205633

